# SQUAT: A web tool to mine human, murine and avian SAGE data

**DOI:** 10.1186/1471-2105-9-378

**Published:** 2008-09-18

**Authors:** Johan Leyritz, Stéphane Schicklin, Sylvain Blachon, Céline Keime, Céline Robardet, Jean-François Boulicaut, Jérémy Besson, Ruggero G Pensa, Olivier Gandrillon

**Affiliations:** 1 Equipe "Bases Moléculaires de l'Autorenouvellement et de ses Altérations", Université de Lyon, F-69622, Université Lyon 1, Villeurbanne, CNRS, UMR5534, Centre de Génétique Moléculaire et Cellualire, Lyon, France; 2 Laboratoire d'InfoRmatique en Image et Systèmes d'information, UMR 5205 CNRS, Bâtiment Blaise Pascal, INSA Lyon, 43 bd du 11 novembre 1918, 69622, Villeurbanne Cedex, France; 3 Pôle Rhône-Alpin de BioInformatique, Université de Lyon, F-69622, Université Lyon 1, Villeurbanne, Lyon, France,

## Abstract

**Background:**

There is an increasing need in transcriptome research for gene expression data and pattern warehouses. It is of importance to integrate in these warehouses both raw transcriptomic data, as well as some properties encoded in these data, like local patterns.

**Description:**

We have developed an application called SQUAT (SAGE Querying and Analysis Tools) which is available at: . This database gives access to both raw SAGE data and patterns mined from these data, for three species (human, mouse and chicken). This database allows to make simple queries like "In which biological situations is my favorite gene expressed?" as well as much more complex queries like: ≪what are the genes that are frequently co-over-expressed with my gene of interest in given biological situations?≫. Connections with external web databases enrich biological interpretations, and enable sophisticated queries. To illustrate the power of SQUAT, we show and analyze the results of three different queries, one of which led to a biological hypothesis that was experimentally validated.

**Conclusion:**

SQUAT is a user-friendly information retrieval platform, which aims at bringing some of the state-of-the-art mining tools to biologists.

## Background

There is an increasing need in transcriptome research for gene expression data and pattern warehouses. One important challenge is to extract meaningful information from transcriptomic data. This is a typical task of Knowledge Discovery from Database (KDD; [1]).

Data generated by Serial Analysis of Gene Expression (SAGE) potentially enclose very useful information on the studied biological systems [2]. This technique is based on the sequencing of short transcript sequences that are assumed to be specific to each transcript. The amount of each tag in SAGE libraries accurately represents the corresponding gene expression level in the original cell population. The aim of our work is to provide a publicly available tool allowing biologists to exploit SAGE data and the patterns they contain.

There are a number of existing tools on the web allowing the querying of SAGE data. Web tools such as SAGEGenie [3] or WebSAGE [4] enable users to perform analysis between two SAGE libraries. This is in line with the fact that most of the statistical analysis tools dedicated to SAGE data are designed to discover a set of tags differentially expressed through two biological situations (see e.g. [5] and [6]). This kind of analysis is interesting but limited. For example, it does not exploit an interesting advantage of SAGE technique: the possibility to perform direct comparisons of expression levels measured from several and heterogeneous experimental conditions [2]. This task is very difficult if not impossible with microarray data (see for example [7]).

Some KDD approaches were tested to mine SAGE data, including global approaches such as clustering [8] or local pattern mining [9,10]. Local pattern discovery techniques such as association rule discovery [11-15] or formal concept extraction [16,17] proved to be useful to mine gene expression data, including SAGE data. A recent review highlights the relevance of mining such local patterns with respect to clustering analyses [9]. For the biologist, a local pattern is an association between some genes displaying specific expression properties and the situations where those genes display such properties. In SQUAT, two types of patterns are made available:

1. Formal concepts, which are the maximal sets of genes over-expressed in the maximal number of situations. This is the reason why over-expression has to be encoded in a binary fashion (over-expressed/not over-expressed: true value for over-expression and a false value otherwise; see [11] and [18] for a discussion of the binarization techniques). Maximal sets of true values are then computed so that neither gene nor situation can be added to the formal concept without introducing a false value.

2. In order to facilitate browsing and to extract noise tolerant patterns, formal concepts can be further aggregated using a hierarchical clustering. This allows the selection of quasi syn-expression groups (QSGs) which are groups of genes that are most of the time over-expressed in a number of biological situations. QSGs have two main advantages compared to formal concepts: noise tolerance and compactness [17]. QSGs were proved to be very useful to reduce the number of patterns and finally improve the interpretation and the selection of potentially interesting information.

Since no integrated web tool was available to mine SAGE data with such approaches, it prevented the biologists from exploring the full potential of these local pattern mining techniques. Therefore we have built SQUAT, a web tool that allow mining of SAGE data using both expression levels and functional information. SQUAT contains multiple information sources including: 1. Gene expression levels such as measured by SAGE in three species; 2. External information related to these genes like their GO category, or their promoter sequences; and 3. More sophisticated types of data resulting from a KDD process, which are either formal concepts or QSGs. These different types of information can be queried either in an autonomous or simultaneous way, virtually allowing an unlimited number of queries to be performed. Three very specific queries illustrating the power of SQUAT are displayed in the Utility section.

## Construction and content

The SQUAT interface is composed of 4 main query types: "Tag/Gene identification", "Promoter search", "Queries on raw SAGE data" and "Queries on formal concepts". A summary of all the SQUAT possibilities is displayed in Table 1. The use of SQUAT is typically an iterative querying process in which the results of a query may be used to perform the following one(s).

**Table 1:** A summary of SQUAT possibilities.

**You have ...**	**You search for...**	**Way to go**
gene name	corresponding tags	Tag/Gene identification -> Gene information search
	NCBI description	Tag/Gene identification -> Gene information search
	aliases	Tag/Gene identification -> Gene information search
	Gene Ontology data	Tag/Gene identification -> Gene information search
	transcript	Tag/Gene identification -> Gene information search
	promoter	Tag/Gene identification -> Gene information search
	SAGE libraries	Queries on raw SAGE data -> SAGE libraries search
	formal concepts/QSG	Queries on formal concepts -> Simple concepts search

tag sequence	corresponding tags	Tag/Gene identification -> Tag-to-gene assignment
	NCBI description	Tag/Gene identification -> Tag-to-gene assignment
	transcript	Tag/Gene identification -> Tag-to-gene assignment
	promoter	Tag/Gene identification -> Tag-to-gene assignment
	Gene Ontology data	Tag/Gene identification -> Tag-to-gene assignment
	SAGE libraries	Tag/Gene identification -> Tag-to-gene assignment
		Queries on raw SAGE data -> SAGE libraries search
	formal concepts/QSG	Queries on formal concepts -> Simple concepts search
	expression sub-matrix	Queries on raw SAGE data -> Expression sub-matrix
	expression in normal and cancer cells	Queries on raw SAGE data -> Expression sub-matrix

Gene Ontology term	corresponding tags	Queries on raw SAGE data -> Gene Ontology search
	corresponding genes	Queries on raw SAGE data -> Gene Ontology search
	description	Queries on raw SAGE data -> Gene Ontology search
	SAGE library	Queries on raw SAGE data -> Gene Ontology search
	formal concepts/QSG	Queries on formal concepts -> Advanced concepts search

SAGE library	corresponding tags	Queries on raw SAGE data -> Tags search
	SAGEmap description	
	formal concepts/QSG	Queries on formal concepts -> Advanced concepts search
	expression sub-matrix	Queries on raw SAGE data -> Expression sub-matrix

nucleotide sequence	reverse sequence	Tag/Gene identification -> Nucleotids sequence handling
	complementary sequence	

global keywords	corresponding tags	Tag/Gene identification -> Gene product finder
	NCBI description	
	Gene Ontology ID/term	Queries on formal concepts -> Advanced concepts search

accession number (RefSeq) or gene description	promoter	Promoter search

### - THE SOFTWARE ARCHITECTURE (Figure 1)

**Figure 1:**
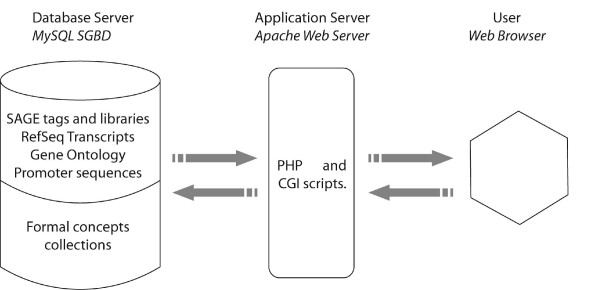
General SQUAT architecture.

The core of SQUAT is based on the MAMP architecture, associating **M**acOS/**A**pache server/**M**ySQL relational database management system/**P**HP programming language.

SQUAT is composed of:

- a relational database, containing SAGE data, a tag identification module (Identitag, see below), a Gene Ontology module, a promoter search module and several collections of patterns extracted from SAGE libraries (see Additional file 1).

- a web interface, dedicated to querying the database and visualizing results in a user-friendly way.

SQUAT is available at: . It is hosted on a Mac OS X server and entirely built using open-source resources. Software versions are Apache 1.3, PHP 4.4.4, MySQL 5.0.2. and Perl 5.8.6. The Perl module BioPerl 1.4 was also used for the cladogram creation and retrieving the promoter sequences in genome assemblies. It was completed with GD 2.0 library to create the whole visualization tool.

### - SQUAT DATABASE

#### - SAGE data

SQUAT contains SAGE data for 3 species: *Homo sapiens*, *Mus musculus *and *Gallus gallus*.

471 human libraries and 494 mouse libraries – including the tags sequences, the tags expression levels and the description of the biological situations of the sample – were downloaded from the NCBI SAGE website [19] as of April 2008.

Regarding the chicken libraries, 13 of them were produced by our group, 4 of which have been published [20,21]. Two chicken libraries have been previously published by another group [22] and were downloaded from the GEO website [23]. The chicken ES cell library was performed within a collaborative framework with Bertrand Pain (INRA, Clermont-Ferrand).

A summary of the SAGE data available in SQUAT is shown in Table 2.

**Table 2:** Current content of the SQUAT website.

	Human	Mouse	Chicken
Number of short SAGE libraries	355	280	13
Number of long SAGE libraries	116	214	2
Total number of different tags	666 189	489 686	105 224
Number of tags used for concepts generation	29 016	29 343	12 345
Number of concepts	314 016	1 104 920	4 691

#### - tag Identification module (Identitag)

SQUAT contains a built-in gene-to-tag assignment function that is performed by an Identitag module [24]. To allow tag identification, transcripts data from RefSeq were downloaded from the NCBI [25]. RefSeq transcripts reference sequences were chosen because it is a non redundant sequence database [26] that therefore permits to minimize the number of multiple sequences identifying one single tag. The following number of RefSeq transcripts were downloaded as of April 2008: chicken: 19257; human: 40091 and mouse: 35135. HUGO gene names were linked to Gene Ontology (see below).

#### - Promoter sequences

The most conservative hypothesis regarding the co-over-expression of a group of genes is that these genes do share common Transcription factor binding sites (TFBS) in their promoter sequences. We therefore decided to incorporate these promoter sequences in SQUAT.

**Figure 2:**
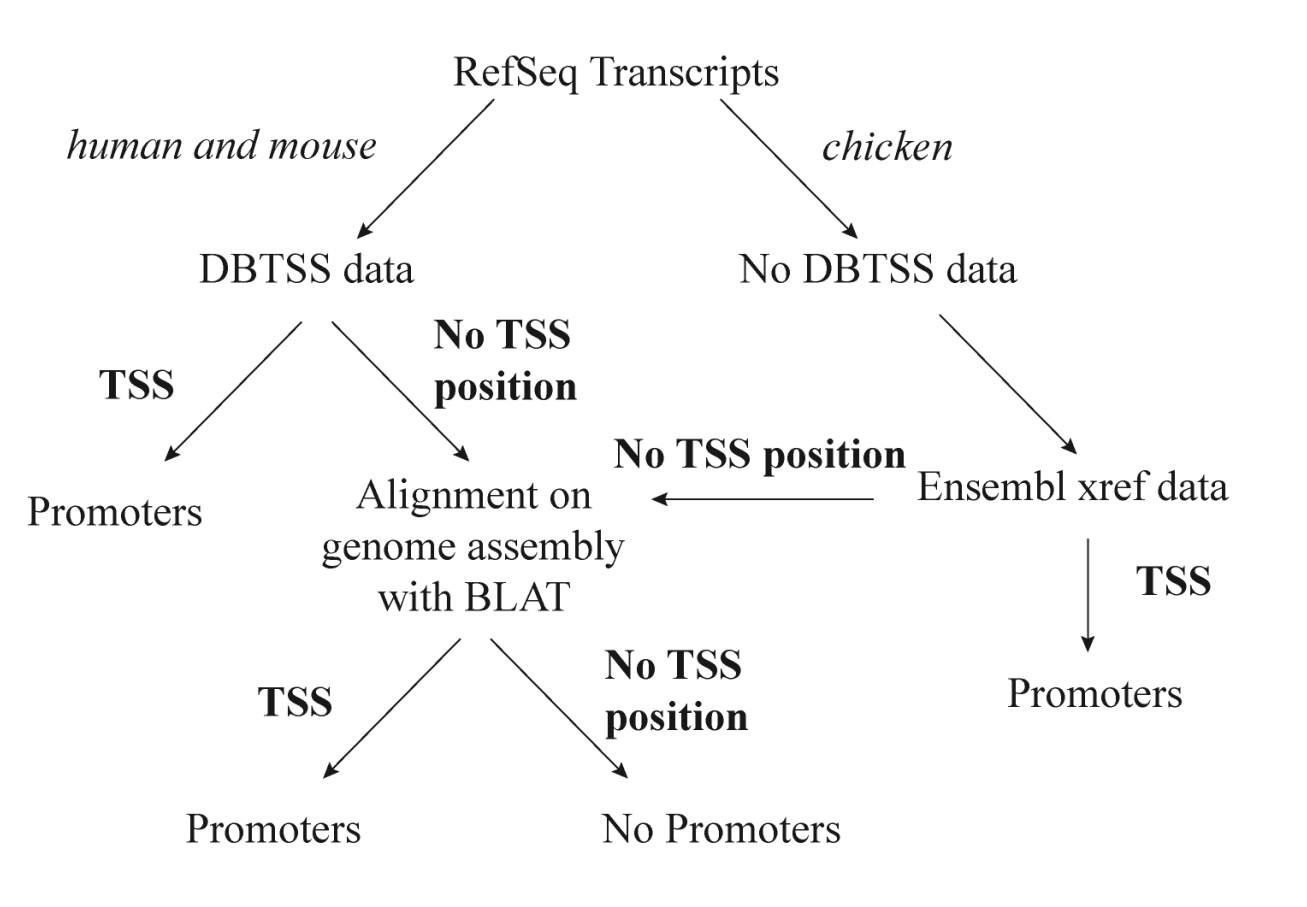
**A schematic view of the pipeline that establishes a link between RefSeq transcripts and their promoter sequence for the three species**. For the human and the mouse, data is available through DBTSS (DataDase of Transcriptional Start Sites; [39]) which provides on one hand the exact RefSeq transcript TSS (Transcriptional Start Sites) position on a genome assembly and on the other hand, when it exists, alternative TSS position for this transcript. DBTSS enables to provide at least one TSS position for 53% of the human transcripts and for 46% of the mouse transcripts. In order to provide TSS positions for the rest of the transcripts, we used BLAT [40]. 83% of the human transcripts and 75% of the mouse transcripts were thereby endowed with a TSS position. Since there is no data available in DBTSS for the chicken, we first used data coming from Ensembl [41] to establish, when possible, the link between the RefSeq transcripts and the Ensembl transcripts. Some rare RefSeq transcripts correspond to several Ensembl transcripts, which confer to our database alternative TSS positions for the chicken as well. Transcripts which could not be linked to Ensembl were also aligned with BLAT on the same version of genome assembly used by Ensembl release. Finally, 85% of chicken RefSeq transcripts have found a TSS position with this pipeline which is close to the value obtained for the two other species.

The location of transcripts on genomes enables us to define the transcription start site (TSS) position and thus, recover the promoter sequence of the genes (Figure 2). SQUAT contains data on the location of the RefSeq transcripts on recent genomes assemblies:

- NCBI build 2.1, WASHUC2 (May 2006) for *Gallus gallus*

- NCBI build 36.1 (March 2006) for *Homo sapiens*

- NCBI build 36 (February 2006) for *Mus musculus*

SQUAT provides the tag-to-TSS relationship, and lets the user decide what region of the genome he wants to consider as a promoter, both 5' and 3' from the TSS. The resulting sequence can then be retrieved.

#### - Gene Ontology

25424 GO terms are available in SQUAT. The March 2008 monthly release of the SQL archive containing GO terms definitions, IDs, associations and gene names, was downloaded from . This set of GO tables was linked to the other SQUAT relational database tables via HUGO gene names (see Additional file 1).

#### - Mined patterns

From the SAGE libraries, one can build 3 gene expression matrices for the 3 species. In order to avoid confusion generated by either unidentified tags, or ambiguous tags (tags mapping to more than one gene), the gene expression matrices were built exclusively from the tags mapping to one (and only one) refSeq transcript (see Table 2 for the number of resulting transcripts).

**Figure 3:**
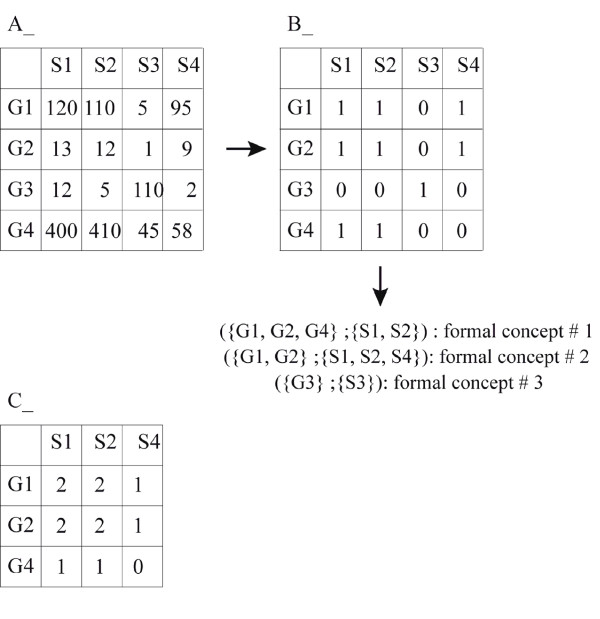
**Gene expression matrix, (A), formal concepts (B) and QSQ (C)**. In A is shown a toy example of a gene expression matrix displaying the level of expression of 4 genes (G1 – G4) in 4 biological situations (S1 – S4). In order to extract formal concepts, one has first to encode some gene expression property. We decided to encode the over-expression by applying the mid-range method [11]. One first defines a threshold per gene (max value – min value)/2 – min value). For the G1 gene, this threshold = 62.5. All expression values below or equal to the threshold are considered null, all values strictly above the threshold are set to 1. This allows to create the binary matrix (B). One then extracts all formal concepts from such a matrix. It consists of a bi-set of genes and situations such that all genes are simultaneously over-expressed in the situations, and such that neither gene nor situation can be added without introducing a null value (those are maximal bi-sets). From the toy example, three formal concepts can be extracted (shown below the B matrix). It is immediately apparent that the two first concepts are closely related. It is therefore tempting to aggregate them, allowing the creation of a Quasi-synexpression group (QSG; [17]) containing three genes and three situations. One possible representation of a QSG is shown in C, the values indicating the number of formal concepts supporting the Gene-to-Situation association.

These matrices can then be mined to extract gene expression patterns. Two types of patterns can be mined through SQUAT: formal concepts and Quasi-synexpression groups (Figure 3). They both are bi-sets associating sets of genes and sets of biological situations.

A formal concept represents a maximal set of genes simultaneously over-expressed in a maximal set of biological situations. This means that all the genes from the concept are simultaneously co-over-expressed in the group of situations, and that one can neither add a gene nor a situation without introducing a false value (a gene not over-expressed in a biological situation). To extract them, SAGE data must first be transformed in a Boolean expression matrix encoding the over-expression property. For the human and murine datasets, we used the Mid-range method which proved its relevancy in previous studies [11,17]. The gene expression matrix dimensions and the amount of correlation in the data [27] generate a huge number of formal concepts. To limit the number of formal concepts, only the formal concepts containing at least 3 tags and 2 libraries were generated for human and murine libraries. Concerning *Gallus gallus*, the whole collection of concepts was created using the three previously described over-expression encoding methods (Mid-range method, Max-xMax and x%Max; [11]) thus allowing more flexibility for the end user to set the over-expression threshold parameter.

Formal concepts were generated using the freely available D-miner algorithm from the BioMiner software [28,29]. SQUAT stores the formal concepts that have been generated from SAGE data matrices for a given species (see Table 2 for the number of concepts).

Due to the exponential growth of the number of extracted patterns with respect to the number of SAGE experiments, storing collections of formal concepts can be difficult. The database design allows an efficient storage and good performances to query the stored patterns.

## Utility

In this section, some queries on the human section of SQUAT are described to illustrate the usefulness and the power of SQUAT. Similar queries can be performed in the murine and avian section.

### 1. Query based on a biological function

Let us assume that the user is interested in the oxygen transport function in human. One question could be: what are the genes related to this function and in which libraries are they over-expressed? This query can be performed by finding all the formal concepts consisting of genes that do contain the GO term "oxygen transport" in their description.

For this we first use the "*Advanced concepts search*" from the "*Queries on formal concepts*" menu which allows to choose precise constraints on concept composition. We first use the ≪*Find an accession number*≫ function in order to obtain the GO number corresponding to oxygen transport. Using one of the two related GO terms (GO:0015671), the previous form is automatically filled. The user can now start a query that returns 51 formal concepts.

**Figure 4:**
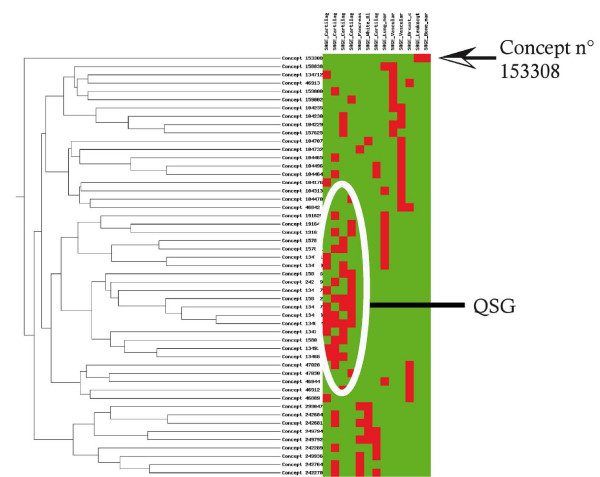
**Hierarchical clustering analysis of the 51 formal concepts.** The concepts shown on the left are represented according to the libraries they contain (shown on top). A red square indicates a library within a concept, a green square a library not within the corresponding concept. From the hierarchical clustering shown on the left, one concept appears to be very different from the rest (n° 153308), and a group of concept appears sufficiently similar to be grouped within a QSG.

These formal concepts can then be clustered using the UPGMA (Unweighted Pair Group Method with Arithmetic mean) algorithm. It is clear that one concept (n° 153308) stands out (Figure 4). When explored in details, it becomes clear that this concept highlights an association between hemoglobin expression (tag sequence: GCAAGAAAGT) and bone marrow-derived libraries. Similarly, formal concepts associating cytoglobin gene over-expression and cartilage-derived libraries are clustered together (Figure 4). The biological relevance of these results is validated by a rapid literature search [30,31].

### 2. Query based on an unknown gene name

One can search for a specific kind of human globin protein, knowing neither its gene symbol nor its related tag sequence. The Tag/gene identification functionality allows the user to find the best input that fits its query, using only a global word. In our example, this query is divided in two quick steps: by using the *Gene product search*, the user can see all the genes products associated with the keyword "globin". SQUAT finds 40 gene products containing this keyword. For each of these genes the HUGO gene name, the RefSeq Id, the GI reference number, a short description and the gene products aliases (if available) are displayed.

We decided to focus on cytoglobin (CYGB). Now that the correct HUGO gene name is known, the *Gene information search *will report the information stored in SQUAT about this gene: gene aliases and a short description as in the previous mode, but also related tag(s), transcript(s) sequences and referring Gene Ontology terms.

In CYGB case, 2 tags are found: CCTGGGTCTC and CAGGTCTCCA. Using ≪*See transcript sequence*≫ it is easy to see that the CCTGGGTCTC tag is the most 3' for this gene and will now be used. Using this tag sequence, we first perform a ≪*Queries on raw SAGE data/SAGE libraries search*≫, showing that this tag was found in 99 situations (representing 21.02% of all the libraries). One can then examine whether there is some homogeneous set of situations where the over-expression of cytoglobin is recorded. For this a "*simple concept search*" was performed. This resulted in the generation of 14 formal concepts comprising 5 libraries. The biological situations can then be examined using the "*graphical mode*" display. Using the "Display library-homogeneous concepts" function, only one concept remains, showing over-expression of cytoglobin in two cartilage chondrosarcoma cell types. No over-expression of cytoglobin has been described yet in chondrosarcomas, and therefore this should be investigated further by biological means.

### 3. Looking for gene expression pattern in normal and cancer cells

We have recently taken an interest in the biological function of the Sca2 gene (HUGO gene name: LY6E; [32]). Since we obtained evidence demonstrating its involvement in the self-renewal of chicken erythroid progenitors [21], we were interested in exploring its expression pattern in human cells. For this we used the sca2 tag (CACTTCAAGG) and found through SQUAT that sca2 is expressed in 328 human SAGE libraries 69.64% of all the libraries, including 295 cancer and 33 normal libraries. Using the "*Expression sub-matrix creation*" menu, we easily demonstrated that sca2 is over-expressed in a high proportion of cancer libraries (mean expression level of 147.75 tags per million, in 295 libraries), mostly in carcinomas and mesotheliomas, as compared with normal libraries (mean expression level of 56,93 tags per million, in 33 libraries). We have confirmed, by quantitative PCR, that sca2 is indeed over-expressed in cancerous human colon and kidney tissues as compared with normal tissues [33].

**Figure 5:**
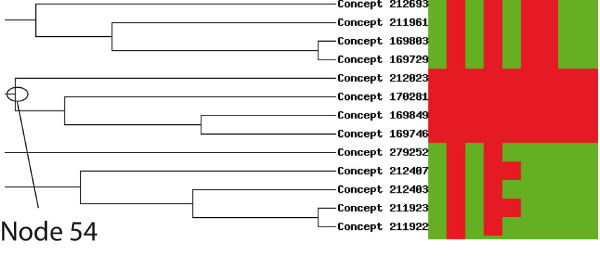
**Hierarchical clustering analysis of the formal concepts associating the Sca2 tag.** Shown is a part of the clustering displaying concepts (on the left) contingent upon the tags they contain (not shown, on top). A red square indicates a tag within a concept, a green square a tag not within the corresponding concept. From the hierarchical clustering shown on the left, one group of concept appears sufficiently similar to be grouped within a QSG, and is extracted using the node 54.

**Figure 6:**
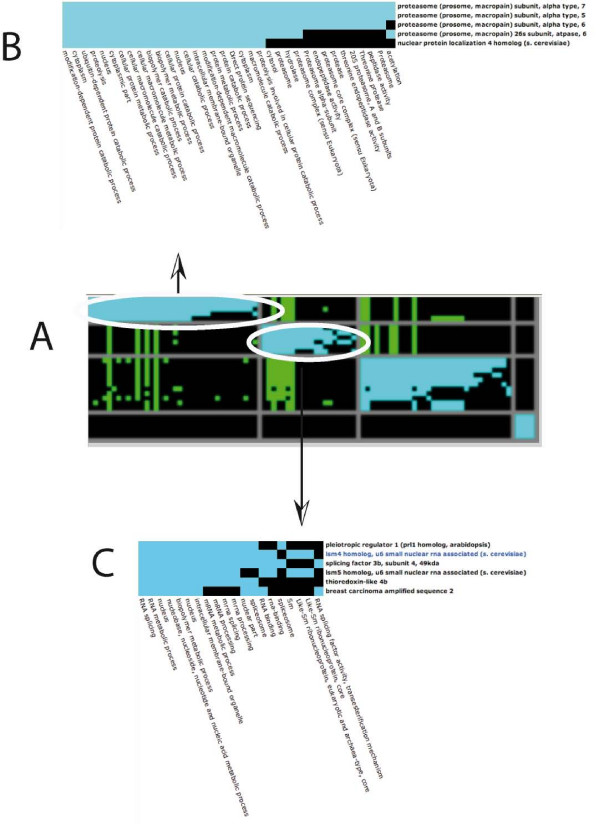
**DAVID analysis of the genes associated in the QSG associated with node 54 (see Figure 5).** In A is shown a graphical representation of the four overrepresented groups obtained using the "Gene Functional Classification" menu with a "high classification stringency" option. The four groups displayed an enrichment of respectively 3.01 times, 2.48 times, 1.21 times and 0.19 times. The genes are displayed in lines and the functional categories in columns. In B and C are shown an enlargement of the first two groups. Blue squares indicate a gene that belongs to a functional category.

Since the function of this gene is largely unknown, we searched with SQUAT for all the genes that are simultaneously over-expressed together with the human sca2 gene in cancer libraries. For this, we performed a "*Query on formal concepts/simple concept search*". This brought back 65 concepts. We then performed a dendrogram representation using the UPGMA algorithm. Looking at the clustering, we decided to explore the Node 54 (Figure 5. Please note that due to the calculation of the cluster and the non exact nature of clustering, different clusterings will be obtained after each trial. Reloading the page will at some point give this representation). By clicking on this node, we have access to the "*Cluster characteristics*≫ section, which shows that the QSG comprises 4 concepts, containing 3 libraries and 194 tags. The 3 libraries are all derived from Breast carcinoma CL MCF7 cells. Furthermore, we can access the ≪*See the cluster composition based on Gene Ontology terms*≫ section. At first sight no major functional category seems to be overrepresented (not shown). We then decided to go one step further, and we downloaded the HUGO names of the genes present within this QSG. We then loaded those names into either the L2L ([34]; results not shown) or the DAVID Database ([35]; Figure 6). The DAVID tool detected within this list 4 groups of genes functionally enriched (Figure 6A). The first group was enriched 3.01 times and its function was mostly associated with the proteasome (Figure 6B). The second group was enriched 2.48 times and its function was mostly associated with the RNA binding activities (Figure 6C).

This raises some interesting hypotheses as to what the function of Sca2 in breast cancer cells might be, requiring experimental investigation.

## Discussion

We have built SQUAT, a database containing SAGE data from three species. Beyond tag sequence and expression levels, SQUAT displays important additional information on tags and on libraries, allowing the end-user to perform sophisticated, iterative queries.

The main difference between SQUAT and existing SAGE repository databases, is the possibility to query both data and patterns extracted from the data in the same process. As such, SQUAT is a first step toward inductive databases (IDB; for recent publications, see [36]). As it stands it is a proof of concept for IDB that may evolve thank's to user feedback.

Based upon our long term effort in mining SAGE data [11,17], we have chosen to implement through SQUAT the possibility to mine formal concepts, that are the maximal set of tags simultaneously co-over-expressed in the maximal number of situations. Since this local pattern technique produces a very large amount of patterns, we also added in SQUAT our recently described clustering strategy leading to QSGs that can be seen as noise-tolerant patterns [17]. This is of special interest in the case of transcriptomic data that are intrinsically noisy, for technical reasons due to the method, as well as for biological causes due to the intrinsic gene expression variability [37]. QSGs are fault-tolerant patterns that can be viewed as formal concepts in which a limited number of exceptions are tolerated: one tolerates that a few genes are not over-expressed in a small subset of situations, in the final synexpression group.

We illustrate here the potential of SQUAT with a set of three queries, starting from a biological function, an unknown gene name, or a known tag.

All together this illustrates the power of the mining tool embedded in SQUAT. This is only a very small part of what can be done using SQUAT. Due to the very large amount of data from different biological situations and the huge amount of patterns extracted from these data, one can only speculate as to the wealth of biological information that SQUAT may bring to the biologists. This is further reinforced by the fact that there are several different ways to query the database.

Several additional resources are planned in the long run.

First, we are in the process of adding a list of Transcription Factor Binding Sites putatively binding to the promoter sequences. Based upon the hypothesis that genes in a formal concept contain common transcription factors, explaining their similar behavior (i.e. over-expression), SQUAT will allow to explore, at the sequence level, putative signature motifs that could explain the molecular basis for the formal concepts (see e.g, [20]). When related to transcription factors binding to these motifs, this will ultimately give hints to build a molecular network incorporating transcription factors and target genes.

Second, we are planning to add orthologous relationship between tags originating from orthologous genes in different species. We have described how tag-to-tag orthologous relationship could be established [24]. This was recently improved (Keime et al., in preparation) and the improved version will be used for SQUAT update. This should constitute a major contribution to the emerging studied field of comparative transcriptomics. This will allow to explore questions like: ≪Can we find groups of genes that are simultaneously over-expressed both in a human tissue and in its murine counterpart?≫.

Another source of external information might be helpful to make sense of specific gene expression pattern that is extracted from the existing bibliography. This can be extracted using text mining methods and added to enrich the queries on the database [27]. This can improve the specificity of some patterns and help the biologists focus on patterns for which the most recent knowledge is available.

An important improvement will also be the possibility to calculate patterns "on the fly", and not, as it presently stands, to query among a set of pre-calculated patterns. The user can specify constraints on these results, but cannot yet modify important parameters such as the over-expression thresholds or the minimal value for the concept size. This should allow much more flexibility in the mining process, although this is out of reach of the current algorithms.

Furthermore, one can also envision to let the user choose among different types of local patterns including association rules [11], emerging patterns (patterns that are characteristic of a given class, like cancer-specific patterns; [38]) or any type of local pattern [9,10].

This will make SQUAT the choice mining tool for all those who want to use local patterns without having the burden to set up the whole analysis process by themselves.

## Conclusion

We have described SQUAT, a database containing different types of data, including raw SAGE expression values, external information sources and local patterns. SQUAT allows to perform both simple and sophisticated queries either simultaneously or independently on the three types of data. Three specific queries have illustrated the power of SQUAT.

## Availability and requirements

SQUAT is available at .

## Authors' contributions

JL designed and encoded a complete version of SQUAT. SS designed and encoded the promoter search part, and integrated it into SQUAT. Both JL, JB and SS participated in the debugging of SQUAT. OG and JL updated the database. SB provided guidance as to the general structure of SQUAT as well as for formal concept visualization and analysis. CK helped integrating Identitag into the database. CR, JB, RGP and JFB wrote the Biominer software, and provided guidance for integrating formal concepts into the database. JL and OG performed and analyzed the data shown in the Utility section. OG designed and coordinated the work. JL, SS, SB and OG participated in writing the manuscript. All authors read and approved the final version of the manuscript.

## Supplementary Material

Additional file 1 SQUAT relational schema. This figures displays the tables and the relation between the table of the SQUAT database. Click here for file

## References

[B1] Maimon O, Rokach L (2005). The Data Mining and Knowledge Discovery Handbook. Springer.

[B2] Velculescu VE, L Zhang, B Vogelstein, KW Kinzler (1995). Serial analysis of gene expression. Science.

[B3] SAGEGenie. http://cgap.nci.nih.gov/SAGE.

[B4] Pylouster J, Senamaud-Beaufort C, Saison-Behmoaras TE (2005). WEBSAGE: a web tool for visual analysis of differentially expressed human SAGE tags. Nucleic  Acids Res.

[B5] Pylouster J, Senamaud-Beaufort C, Saison-Behmoaras TE (2005). WEBSAGE: a web tool for visual analysis of differentially expressed human SAGE tags. Nucleic Acids Res.

[B6] Romualdi C, Bortoluzzi S, SM W (2005). Web tools for statistical Analysis of SAGE data. SAGE: current technologies and applications.

[B7] Severgnini M, Bicciato S, Mangano E, Scarlatti F, Mezzelani A, Mattioli M, Ghidoni R, Peano C, Bonnal R, Viti F, Milanesi L, De Bellis G, Battaglia C (2006). Strategies for comparing gene expression profiles from different microarray platforms: application to a case-control experiment. Anal Biochem.

[B8] Ng TR, Sander J, Sleumer M (2001). Hierarchical Cluster Analysis of SAGE Data for Cancer Profiling. workshop on Data Mining in BioInformatics with SIGKDD '01.

[B9] Madeira SC, Oliveira AL (2004). Biclustering algorithms for biological data analysis: a survey. IEEE/ACM Transactions on Computational Biology and Bioinformatics.

[B10] Prelic A, Bleuler S, Zimmermann P, Wille A, Buhlmann P, Gruissem W, Hennig L, Thiele L, Zitzler E (2006). A systematic comparison and evaluation of biclustering methods for gene expression data. Bioinformatics.

[B11] Becquet C, Blachon S, Jeudy B, Boulicaut JF, Gandrillon O (2002). Strong-association-rule mining for large-scale gene-expression data analysis: a case study on human SAGE data. Genome Biol.

[B12] Creighton C, Hanash S (2003). Mining gene expression databases for association rules. Bioinformatics.

[B13] Elati M, Radvanyi F, Rouveirol C (2005). Mining transcriptional regulation from expression data. Actes des Journées Ouvertes de Biologie Informatique et Mathématiques (JOBIM): 2005; Lyon.

[B14] Georgii E, Richter L, Ruckert U, Kramer S (2005). Analyzing microarray data using quantitative association rules. Bioinformatics.

[B15] Li J, Liu H, Downing JR, Yeoh AE, Wong L (2003). Simple rules underlying gene expression profiles of more than six subtypes of acute lymphoblastic leukemia (ALL) patients. Bioinformatics.

[B16] Rioult F, Robardet C, Blachon S, Crémilleux B, Gandrillon O, Boulicaut JF (2003). Mining concepts from large SAGE gene expression matrices. 2nd Int Workshop Knowledge Discovery in Inductive Databases KDID'03 co-located with ECML-PKDD 2003: September 22 2003; Cavtat-Dubrovnik (Croatia).

[B17] Blachon S, Pensa RG, Besson J, Robardet C, Boulicaut J-F, Gandrillon O (2007). Clustering formal concepts to discover biologically relevant knowledge from gene expression data. Silico Biol.

[B18] Pensa R, Boulicaut JF (2005). Boolean property encoding for local set pattern discovery: an application to gene expression data analysis. Local Pattern Detection Springer-Verlag LNAI.

[B19] SAGE N. ftp://ftp1.nci.nih.gov/pub/SAGE/.

[B20] Bresson C, Keime C, Faure C, Letrillard Y, Barbado M, Sanfilippo S, Benhra N, Gandrillon O, Gonin-Giraud S (2007). Large-scale analysis by SAGE reveals new mechanisms of v-erbA oncogene action. BMC Genomics.

[B21] Damiola F, Keime C, Gonin-Giraud S, Dazy S, Gandrillon O (2004). Global transcription analysis of immature avian erythrocytic progenitors: from self-renewal to differentiation. Oncogene.

[B22] Wahl MB, Caldwell RB, Kierzek AM, Arakawa H, Eyras E, Hubner N, Jung C, Soeldenwagner M, Cervelli M, Wang YD, Liebscher V, Buerstedde JM (2004). Evaluation of the chicken transcriptome by SAGE of B cells and the DT40 cell line. BMC Genomics.

[B23] GEO http://www.ncbi.nlm.nih.gov/geo/.

[B24] Keime C, Damiola F, Mouchiroud D, Duret L, Gandrillon O (2004). Identitag, a relational database for SAGE tag identification and interspecies comparison of SAGE libraries. BMC Bioinformatics.

[B25] National Center for Biotechnology Information. http://www.ncbi.nlm.nih.gov/.

[B26] Pruitt KD, Tatusova T, Maglott DR (2007). NCBI reference sequences (RefSeq): a curated non-redundant sequence database of genomes, transcripts and proteins. Nucleic Acids Res.

[B27] Klema J, Soulet A, Crémilleux B, Blachon S, Gandrillon O (2006). Mining Plausible Patterns from Genomic Data. 19th IEEE International Symposium on Computer-Based Medical Systems: 2006; Salt Lake City, Utah.

[B28] BioMiner. http://liris.cnrs.fr/dmidb/BioMiner/.

[B29] Besson J, Robardet C, Boulicaut J-F, Rome S (2005). Constraint-based concept mining and its application to microarray data analysis. Intelligent Data Analysis.

[B30] Hankeln T, Wystub S, Laufs T, Schmidt M, Gerlach F, Saaler-Reinhardt S, Reuss S, Burmester T (2004). The cellular and subcellular localization of neuroglobin and cytoglobin – a clue to their function?. IUBMB Life.

[B31] Ostojic J, Sakaguchi D, de Lathouder Y, Hargrove M, Trent J, Kwon Y, Kardon R, Kuehn M, Betts D, Grozdanic S (2006). Neuroglobin and cytoglobin: oxygen-binding proteins in retinal neurons. Invest Ophthalmol Vis Sci.

[B32] Fleming TJ, Malek TR (1994). Multiple glycosylphosphatidylinositol-anchored Ly-6 molecules and transmembrane Ly-6E mediate inhibition of IL-2 production. J Immunol.

[B33] Bresson C, Gandrillon O, Gonin-Giraud S (2008). sca2: a new gene involved in the self-renewal of erythroid progenitors. Cell Proliferation.

[B34] Newman JC, Weiner AM (2005). L2L: a simple tool for discovering the hidden significance in microarray expression data. Genome Biol.

[B35] Dennis G, Sherman BT, Hosack DA, Yang J, Gao W, Lane HC, Lempicki RA (2003). DAVID: Database for Annotation, Visualization, and Integrated Discovery. Genome Biol.

[B36] Boulicaut JF (2004). Inductive databases and multiple uses of frequent itemsets: the cInQ approach. Database support for Data Mining Applications – Discovering Knowledge with Inductive Queries.

[B37] Kaern M, Elston TC, Blake WJ, Collins JJ (2005). Stochasticity in gene expression: from theories to phenotypes. Nat Rev Genet.

[B38] Soulet A, Crémilleux B, Rioult F (2005). Condensed Representation of EPs and Patterns Quantified by Frequency-Based Measures. Lecture Notes in Computer Science.

[B39] Database of Transcriptional Start Sites. http://dbtss.hgc.jp.

[B40] Kent WJ (2002). BLAT – the BLAST-like alignment tool. Genome Res.

[B41] Ensembl. http://www.ensembl.org.

