# Supplementary figures and images for: SQUAT: A web tool to mine human, murine and avian SAGE data
Source: BMC Bioinformatics. 2008 Sep 18;9:378. doi: 10.1186/1471-2105-9-378 (PMC2567996; doi:10.1186/1471-2105-9-378)

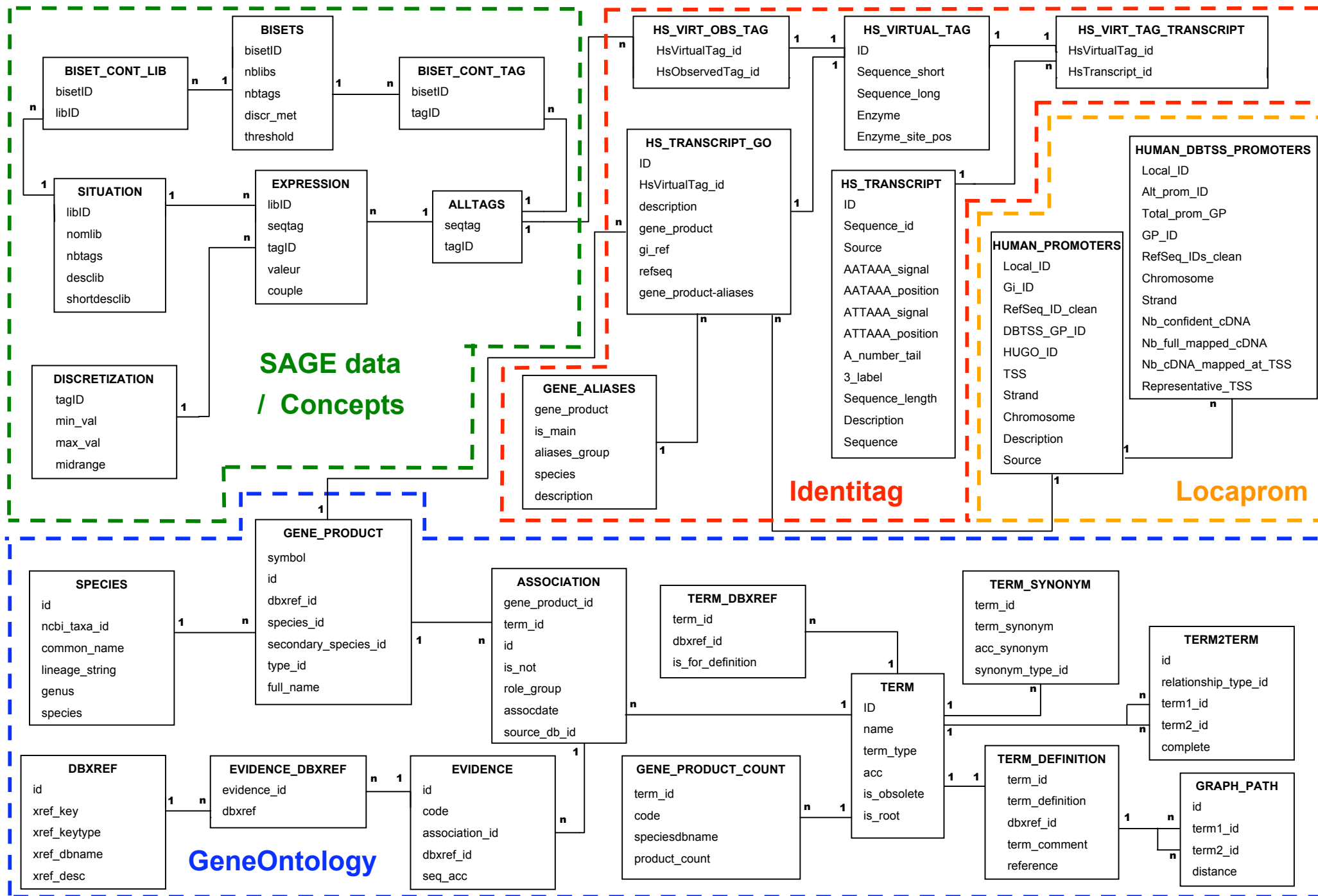

Supplement: Additional file 1 — SQUAT relational schema. This figures displays the tables and the relation between the table of the SQUAT database. [file 1471-2105-9-378-S1.pdf]
